# Description, Taxonomy, and Comparative Genomics of a Novel species, *Thermoleptolyngbya sichuanensis* sp. nov., Isolated From Hot Springs of Ganzi, Sichuan, China

**DOI:** 10.3389/fmicb.2021.696102

**Published:** 2021-09-10

**Authors:** Jie Tang, Liheng Li, Meijin Li, Lianming Du, Md Mahfuzur R. Shah, Michal M. Waleron, Malgorzata Waleron, Krzysztof F. Waleron, Maurycy Daroch

**Affiliations:** ^1^Antibiotics Research and Re-evaluation Key Laboratory of Sichuan Province, Sichuan Industrial Institute of Antibiotics, Chengdu University, Chengdu, China; ^2^School of Environment and Energy, Peking University Shenzhen Graduate School, Shenzhen, China; ^3^Department of Pharmaceutical Microbiology, Faculty of Pharmacy, Medical University of Gdańsk, Gdańsk, Poland; ^4^Department of Plant Protection and Biotechnology, Intercollegiate Faculty of Biotechnology, University of Gdańsk and Medical University of Gdańsk, Gdańsk, Poland

**Keywords:** *Thermoleptolyngbya*, 16S rRNA, 16S-23S ITS, comparative genomics, thermophilic cyanobacterium, Oculatellaceae

## Abstract

Thermoleptolyngbya is a newly proposed genus of thermophilic cyanobacteria that are often abundant in thermal environments. However, a vast majority of *Thermoleptolyngbya* strains were not systematically identified, and genomic features of this genus are also sparse. Here, polyphasic approaches were employed to identify a thermophilic strain, PKUAC-SCTA183 (A183 hereafter), isolated from hot spring Erdaoqiao, Ganzi prefecture, China. Whole-genome sequencing of the strain revealed its allocation to *Thermoleptolyngbya* sp. and genetic adaptations to the hot spring environment. While the results of 16S rRNA were deemed inconclusive, the more comprehensive polyphasic approach encompassing phenetic, chemotaxic, and genomic approaches strongly suggest that a new taxon, *Thermoleptolyngbya sichuanensis* sp. nov., should be delineated around the A183 strain. The genome-scale phylogeny and average nucleotide/amino-acid identity confirmed the genetic divergence of the A183 strain from other strains of *Thermoleptolyngbya* along with traditional methods such as 16S-23S ITS and its secondary structure analyses. Comparative genomic and phylogenomic analyses revealed inconsistent genome structures between *Thermoleptolyngbya* A183 and O-77 strains. Further gene ontology analysis showed that the unique genes of the two strains were distributed in a wide range of functional categories. In addition, analysis of genes related to thermotolerance, signal transduction, and carbon/nitrogen/sulfur assimilation revealed the ability of this strain to adapt to inhospitable niches in hot springs, and these findings were preliminarily confirmed using experimental, cultivation-based approaches.

## Introduction

Cyanobacteria are widely distributed microorganisms in various ecological niches due to abundant features allowing extensive adaptations. In particular, cyanobacterial populations existing in thermal environments have attracted increased interests in light of a crucial role in energy metabolism and matter cycling in ecosystems (Amin et al., 2017). Among the cyanobacteria communities, strains morphologically assigned to genus *Leptolyngbya* are often reported to be prosperous in many thermal environments (Mackenzie et al., 2012; Amarouche-Yala et al., 2014; Tang et al., 2018b; Strunecký et al., 2019).

Identification of *Leptolyngbya*-like strains has been controversial because of their simple morphology, lacking significant discrimination. The heterogeneity of *Leptolyngbya* has been questioned since the establishment of this genus (Bruno et al., 2009). The genus *Leptolyngbya* has been recognized as polyphyletic (Johansen et al., 2011; Perkerson et al., 2011), and there are strong recommendations to conduct a taxonomic reevaluation of this genus. In light of limited information provided by cell morphology investigations of trichomes, genetic and molecular techniques have been applied to facilitate the establishment of correct taxonomy. The 16S rRNA gene has been proposed as a universal DNA barcoding marker for species-level identification of bacterial isolates, and as a complement to morphology-based taxa identification (Yarza et al., 2014). However, in some cases, the 16S rRNA gene cannot resolve cyanobacterial phylogeny at the species level (Niclas et al., 2010; Eckert et al., 2014). An additional genomic locus, 16S-23S intergenic spacer (ITS), has been used for cyanobacterial systematics. It has been applied for the construction of phylogenetic trees and through the analysis of secondary structures of 16S-23S ITS regions (Johansen et al., 2011). Numerous studies confirmed the integrated approach of analyzing 16S rRNA gene phylogeny and 16S-23S ITS secondary structure to be useful and robust for complex cyanobacterial taxonomy, as in the case of the species or genera within the family *Leptolyngbyaceae* (Komarek et al., 2014; Debnath et al., 2017; Shalygin et al., 2020). In light of these examples, the application of a polyphasic approach for cyanobacteria identification is crucial (Komárek, 2016, 2018).

Recently, *Thermoleptolyngbya*, a cryptogenus newly delineated using 16S rRNA gene and 16S-23S ITS, emerged from strains originally grouped into *Leptolyngbya* and was morphologically characterized by filaments, isopolar trichomes surrounded by a thin colorless sheath, and parietal thylakoids (Sciuto and Moro, 2016). Two species were ascribed to this new taxon: *T. albertanoae* and *T. oregonensis*. Still, the majority of strains ascribed to *Thermoleptolyngbya* were not systematically identified, and 16S rRNA gene sequences were exclusively used for their taxonomic recognition. As such, the genus requires expanded sequence information to complement the 16S rRNA gene taxonomy by multiple-locus sequence analysis (MLSA) or on the whole genome level. The widened sequence space, especially about the underrepresented members of the genus, is required to guide phylogenetic and taxonomic studies and eventual reclassification. In addition, the acquisition of a complete genome may provide new insights into the genomic features of genus *Thermoleptolyngbya*, particularly the survival mechanism in thermal conditions from a genomic perspective.

Strain A183, originally isolated from Erdaoqiao hot springs (Tang et al., 2018b) in Ganzi Prefecture, Sichuan Province, China, was previously reported as a potential new species (Tang et al., 2018a) and was used for taxonomic reevaluation in the current study. The morphological and molecular data were collected for this thermophilic *Thermoleptolyngbya*. This work aimed to provide deeper insights into the taxonomy and genomic features of *Thermoleptolyngbya* strains. Characterization in terms of morphology, physiology, and phylogeny was achieved by microscopic, experimental, and molecular analysis.

## Materials and Methods

### *Thermoleptolyngbya sichuanensis* sp. nov. A183: Origins, Cultivation, and Basic Physiological Assessment

The stock culture of strain A183, cryopreserved for over 2 years as 10% DMSO in BG11 stock in −80°C, was used to establish the final precultures for experiments, essentially as described by Tang et al. (2018a). The shake-flask cultures were grown without carbon supplementation in a shaking incubator in BG11 medium at 45°C, 100 rpm, under a photoperiod of 16-h light (45 μmol m^–2^ s^–1^) and 8-h darkness. Cells were subcultured every 2 weeks until the growth reached the logarithmic phase (OD_685__nm_ of 1.0–1.5). Cells were subcultured by resuspending 2% volume of centrifuged cells into a 100-ml fresh medium in 250-ml conical flasks sealed with a perforated sealing film. The strain was initially denoted and deposited in Peking University Algae Collection as PKUAC-SCTA183 has also been deposited in the Freshwater Algae Culture Collection at the Institute of Hydrobiology (FACHB-collection) with accession number FACHB-2491.

The assessment of the capacity of the strain for nitrate, nitrite, and urea utilization was performed as described previously (Liang et al., 2019). Briefly, strains were cultivated in a nitrogen-free BG-11 medium supplemented with different nitrogen sources up to a concentration: 17 mM NaNO_2_, 85 mM NaNO_3_, 3 mM urea. The effect of sulfates and sulfites on the cultivation of the strain has been tested with the addition of 10 mM Na_2_SO_4_ and 10 mM NaHSO_3_ to the BG-11 medium lacking sulfur, respectively. The growth parameters have been tested against the same strain grown in a standard BG-11 medium. The ability of the strain to fix molecular nitrogen has been performed in the course of 72 h using the acetylene reduction method according to the previously described methodology (Chen et al., 1998).

### Microscopic Analysis

The isolated cyanobacterium was investigated using light microscopy LM, DP72; (Olympus, Tokyo, Japan). Approximately 5 μl of culture was dropped on the microscopy slide and observed under 400× magnification. The images were captured using a U-TV0.63XC camera (Olympus). Scanning electron microscopy (SEM) was performed as follows: cells were washed gently with PBS (Servicebio, Boston, MA, United States, G0002), and fixed for 2 h in fixation solution (Servicebio, G1102). Subsequently, the cells were postfixed with 1% OsO_4_ (Ted Pella Inc., Redding, CA, United States) in 0.1 M phosphate buffer (pH 7.4) for 1–2 h at room temperature. The fixed material was dehydrated in a graded ethanol series (30–100%) (Sinopharm, Shanghai, China) and isoamyl acetate (Sinopharm, Shanghai, China) for 15 min and dried with Critical Point Dryer (Quorum, Laughton, United Kingdom, K850). Specimens were then attached to the metallic stubs using carbon stickers and sputter-coated with gold for 30 s. Coated samples were examined directly under the scanning electron microscope (Hitachi, Tokyo, Japan, SU8100). For transmission electron microscopy (TEM), the fixation solution G1102 (Servicebio) was added to the isolated cells. The cells were subsequently pelleted and resuspended in the fresh fixation solution. Cooled 1% agarose solution was mixed with the cells, and the agarose blocks were post-fixed with 1% OsO_4_ in 0.1 M phosphate buffer (pH 7.4) for 2 h. Then cells were dehydrated as described above for SEM and embedded in pure EMBed 812 resins 90529-77-4 (SPI, West Chester, PA, United States). Embedded cells were incubated in a 65°C oven for more than 48 h to complete polymerization. The sections were cut to 60–80 nm thin layers using the ultra-microtome Leica EM UC7 (Leica, Wetzlar, Germany), stained with 2% uranium acetate saturated alcohol solution and lead citrate for 8 min, and examined using TEM (Hitachi, HT7800).

### Genome Sequencing and Assembly

Genomic DNA of strain A183 was extracted and purified using a bacterial genomic DNA isolation kit (Generay, Shanghai, China) according to the manufacturer’s instructions. Purified genomic DNA was subjected to 1% agarose gel electrophoresis for the analysis of its integrity and assessed spectrophotometrically with Nanophotometer (Impeln) to determine the DNA concentration and optical purity. The whole-genome sequencing of A183 strain was performed using a hybrid sequencing strategy combining PacBio long reads and Illumina short reads. For Illumina sequencing, the libraries were generated using NEBNext^®^ Ultra^TM^ DNA Library Prep Kit for Illumina (NEB, Ipswich, MA, United States) following manufacturer’s recommendations, and index codes were added to attribute sequences to the sample, as the DNA sample was fragmented by sonication to a size of 350 bp. The short read of A183 was sequenced using Illumina NovaSeq PE150 at the Beijing Novogene Bioinformatics Technology Co., Ltd. (Beijing, China). For PacBio sequencing the DNA library with an insert size of 10 kb was constructed and sequencing was performed with P6-C4 chemistry according to manufacturer’s recommendations. Two SMRT cells were used for PacBio sequencing and yielded 57,735 adapter-trimmed reads (subreads) with an average read length of approximately 9 kb, corresponding to 94-fold coverage. Illumina NovaSeq sequencing of strain A183 generated a total of 6,810,074 filtered paired-end reads (clean data), providing approximately 185-fold coverage of the genome. These clean data were assembled into contigs using MaSuRCA v. 3.3.9 with default parameters (Zimin et al., 2013), finally generating a single contig. The genome obtained was mapped with Illumina reads using BWA v0.7.17 (Li and Durbin, 2009) and then Pilon v1.23 (Walker et al., 2014) to correct any assembly and sequence errors.

### Phylogenetic Reconstruction

Sequences of the 16S rRNA gene and 16S-23S ITS were extracted from A183 strain genome for phylogenetic analysis. Reference sequences of cyanobacteria were also retrieved from GenBank through BLAST search for 16S rRNA gene and 16S-23S ITS dataset construction, respectively. Multiple alignments of sequences were generated with Muscle incorporated in Mega7 (Kumar et al., 2016). Alignments were subjected to manual editing where necessary. Sequences of each alignment were trimmed to the same length.

Phylogenetic trees of 16S rRNA and 16S-23S ITS sequence datasets were reconstructed using Maximum-Likelihood (ML), Maximum-Parsimony (MP), and Neighbor-Joining (NJ) methods, respectively. ML phylogenetic analyses were both carried out using PhyML version 3.0 (Guindon et al., 2010), and the substitution models were selected based on the Akaike information criterion (AIC) by Model Selection function implemented in PhyML (Vincent et al., 2017). The NJ trees were both constructed using the General Time Reversible (GTR) model implemented in Mega7. Non-parametric bootstrap tests (1000 replications) were applied to evaluate the robustness of tree topologies.

The phylogenomic relationship between A183 and focus taxa was inferred using the concatenated sequences from single-copy genes shared by all the genomes. Single-copy genes were identified by OrthoMCL (Li et al., 2003), concatenated using a customized Perl script and aligned by MAFFT v7.453 (Standley, 2013). The ML genomic tree was constructed by IQ-TREE v2.1.3 (Minh et al., 2020) using the substitution model selected by ModelFinder implemented in IQ-TREE. Tree topology was assessed by UltraFast Bootstrap (1000 replicates) (Hoang et al., 2018). The strains from the family *Leptolyngbyaceae* were used to root the tree.

### Secondary Structure Prediction

The tRNAs presented in 16S-23S ITS sequences were predicted by tRNAscan-SE v1.3.1 (Lowe and Eddy, 1997). The conserved domains (D1-D1’, D2, D3, boxA, and D4) and the variable regions (V2, boxB, and V3) of 16S-23S ITS were detected as reported by Iteman et al. (2000). The secondary structures of these DNA fragments were individually folded by Mfold web server (Zuker, 2003). Except for the use of the structure draw mode untangle with loop fix, default conditions in Mfold were used in all cases.

### Genome Annotation and Comparative Genome Analysis

The genome of A183 strain was annotated using a modified pipeline previously established by Tang et al. (2019). Briefly, gene prediction and annotation were automatically performed using the NCBI prokaryotic genome annotation pipeline (Pruitt et al., 2009), and further using the RAST annotation system to minimize poor calls. The insertion sequence (IS) was detected and annotated by ISsaga (Varani et al., 2011). Prophage regions were predicted by PHASTER (Arndt et al., 2016). CRISPR loci were detected using CRISPRCasFinder server (Grissa et al., 2007). The protein sequences predicted by RAST were aligned against the NCBI non-redundant database using BLASTP with an *E*-value cut-off of 1e-5. The alignment results were imported into BLAST2GO V5.2.5 (Conesa et al., 2005) for GO term mapping. The results of BLAST2GO analysis were submitted to the WEGO (Ye et al., 2006) for GO classification under the biological process, molecular function, and cellular component ontologies. The circular plot of the A183 genome was generated by Circos v0.68 (Krzywinski et al., 2009).

The genome of the *Thermoleptolyngbya* sp. strain O-77 (AP017367) was used for comparative genomic analysis with strain A183 (CP053661). The genome sequence was also subjected to the annotation pipeline mentioned above to keep all the data analyzed under the same criteria. To compare the gene context, all-against-all BLASTP alignments were performed using the following thresholds: *E*-value cut-off of 1e-5 and 30% identity, and the best hit of alignments was selected. Orthologous genes were identified with the bidirectional best hit (BBH) criterion (Brilli et al., 2010).

The whole-genome average nucleotide identity (ANI) and average amino acid identity (AAI) between genomes were calculated using the ANI/AAI calculator with default settings^1^. Only genomes with near completeness (≥90%) and low contamination (<5%) were retrieved from NCBI for ANI/AAI analysis.

## Results and Discussion

### Morphological Investigation

**FIGURE 1:**
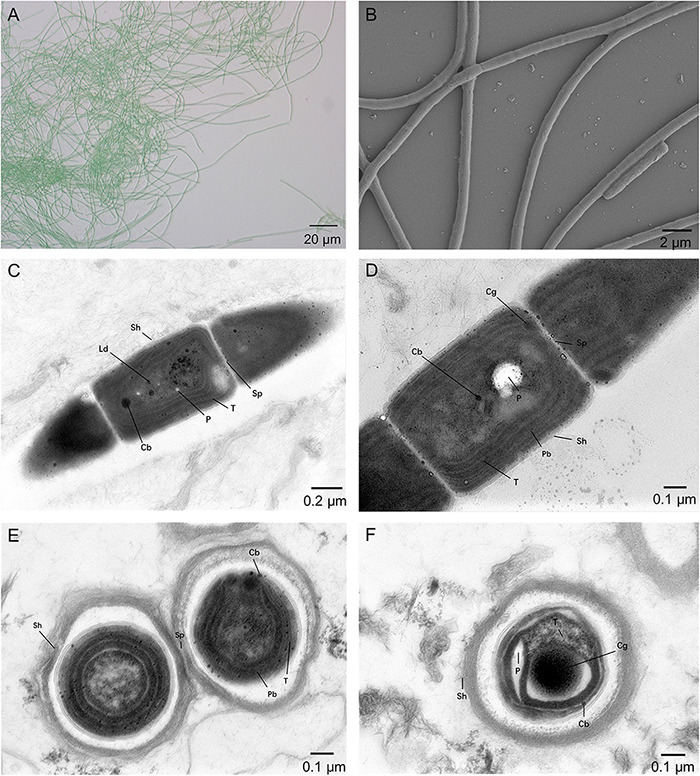
Micrographs of A183. **(A)** Light microscopy image. **(B)** SEM image. **(C–F)** TEM images. Cb, carboxysome; Cg, cyanophycin granule; Cn, cyanophycin granule; Ld, lipid droplet; P, polyphosphate body; Pb, phycobilisome; Sh, sheath; Sp, septum; T, thylakoid membrane. Magnifications: 400× **(A)**, 5,000× **(B)**, 8,000× **(C)**, and 12,000× **(D–F)**.

The cell morphology of the A183 strain indicated by light microscopy revealed straight, wavy, and occasionally bent trichomes (Figure 1A). The SEM and TEM (Figures 1A–D) showed that trichomes of strain A183 were unbranched and composed of 80–120 elongated barrel-shaped cells, 1.30–1.60 μm in length and 1.05–1.10 μm in width. Constrictions were observed at the cross-walls of cells (Figures 1B–D). Individual cells of the filaments were divided by centripetal invagination of the cell wall (Figure 1E). Intracellular connections between vegetative cells were not observed (Figure 1D). The TEM analysis also exhibited that the three to five thylakoid layers were located in parallel at the inner periphery of cells (Figures 1C,D) and can be described as parietal according to recent classification (Mareš et al., 2019). Sheath, septum, phycobilisome, carboxysomes, cyanophycin granule, and polyphosphate bodies were present in the cytoplasm (Figures 1C–F), and small lipid droplets were also observed (Figure 1C).

The morphological characteristics of A183 strain showed certain similarity to that of other *Thermoleptolyngbya* strains (Sciuto and Moro, 2016). The morphological description of all strains belonging to the genus is summarized in Table 1. Strain A183, together with strains ETS-08 and PCC 8501, were all blue-green filamentous cells and exhibited no vesicles. The sheath of A183 and PCC 8501 was unlayered, while ETS-08 showed a multilayered sheath. There were granules observed at the cross walls of A183 and ETS-08 but no of PCC 8501. This observation reinforces the claim that on the morphology level alone, it is impossible to make any final taxonomic conclusions.

**TABLE 1:** Morphological features of known *Thermoleptolyngbya* strains.

**Strain**	**Ecology**	**Cell width (μm)**	**Cell length (μm)**	**Sheath**	**Granule**	**Color**	**Thylakoids No.**	**References**
A183	Hot spring	1.05–1.10	1.30–1.60	Unlayered	Present	Blue-green	3–5	This work
ETS-08	Thermal mud	<1	1.5–3	Multi-layered	Present	Blue-green	3–4	Sciuto and Moro, 2016
PCC 8501	Hot spring	0.8–1.8	2–6.5	Unlayered	Absent	Blue-green	4–6	Sciuto and Moro, 2016

### Physiological Characteristics of Strain A183

Basic physiological characterization of the strain has been assessed by monitoring its growth with various modifications of the BG-11 medium (Table 2). The strain was capable to actively grow using externally added sodium bicarbonate up to a concentration of 0.5 M. This indicates that bicarbonate transporters, typical for many cyanobacteria are also active in A183. Physiological testing of sulfur compounds revealed that the strain responds positively to 10 mM sulfate concentration in the growth medium but negatively to a similar concentration of sulfite. This is similar to previous findings concerning *Thermosynechococcus* E542 (Liang et al., 2019). Analysis of the utilization of various nitrogen sources indicates that the strain is capable of using nitrate and urea as nitrogen sources. The latter promotes the growth of the strain at 3-mM concentrations and inhibits at 6 mM. The A183 strain is also capable of diazotrophy. Acetylene reduction proxy assay revealed that similar to other *Thermoleptolyngbya* strains (Yoon et al., 2017), the A183 possesses a functional nitrogenase capable of molecular nitrogen fixation. In a course of a 72-h-long assay conditions, the cells exhibited nitrogenase activity; the activity was highest in the first 24 h of the assay and then plateaued (Supplementary Figure 1).

**TABLE 2:** The effect of various carbon and nitrogen sources on the growth of *Thermoleptolyngbya sichuanensis* A183.

**Growth medium composition**								
BG-11	100 mM NaHCO_3_	300 mM NaHCO_3_	500 mM NaHCO_3_	85 mM NaNO_3_	3 mM urea	6 mM urea	10 mM Na_2_SO_4_	10 mM NaHSO_3_
+	+++	++	=	+++	+	–	+	–

*“+” Means that A183 can grow, and the more “+”, the better the growth; “–” means that it cannot grow; “ = ” means no change in growth with respect to BG-11 control.*

### General Features of Strain A183 Genome

**FIGURE 2:**
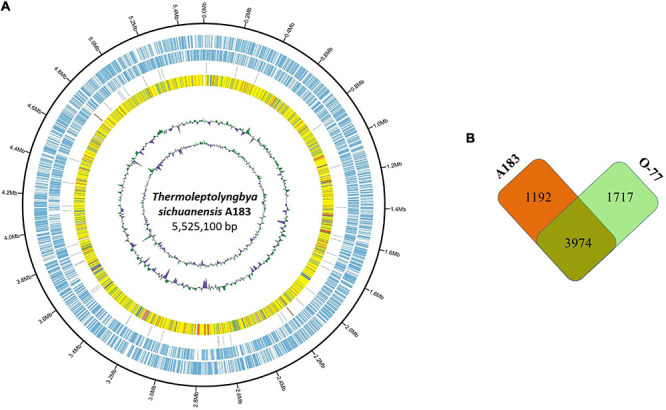
Comparison between *Thermoleptolyngbya* strains. **(A)** Circular plot of A183 genome. Rings are as follows (outer-inner): CDS on plus strand; CDS on minus strand; rRNA (orange) and tRNA (blue); the shared amino acid identities of BLASTP alignments with O-77; the last two circles represent GC content and GC skew both calculated for a 10-kb window with 1-kb stepping. The color scheme for the heat map of orthologs is as follows: yellow, orthologs ≥ 90% identity; blue, 80–90% identity; green, 70–80% identity; red, 50–70% identity; orange, 30%–50% identity. **(B)** Number of shared and specific genes between strains.

The combined assembly of PacBio and Illumina sequencing data successfully generated the complete genome of strain A183. The genome (Figure 2A) comprises a single circular chromosome with a size of 5,525,100 bp (GC content, 56.38 mol%) and no plasmid. Gene prediction and annotation of strain A183 resulted in 5,166 protein-coding sequences (CDS), approximately half (50.3%) of which were identified as hypothetical proteins. Functional distribution on GO categories of all CDS identified was summarized in Supplementary Figure 2. Two ribosomal RNA (*rrn*) operons were detected and 45 tRNA genes were predicted in the A183 chromosome (Table 3).

**TABLE 3:** Genome features of *Thermoleptolyngbya* strains A183 and O-77.

	**A183**	**O-77**
Accession number	CP053661	AP017367
Isolation source	Hot spring, Sichuan Province, China	Hot spring, Kumamoto, Japan
Temperature	40.8°C	Not specified (45–80°C range)
Size (bp)	5,525,100	5,480,261
Chromosome	1	1
GC content (mol%)	56.4	55.9
rRNA operons	2	2
tRNA	45	45
Number of subsystems	266	272
Coding sequences	5166	5691

In the A183 chromosome, 186 ISs representing 28 different IS families were identified. The most frequently observed IS type was the ISKra4 family (30.11%), followed by the IS630 family (26.88%) and IS4 family (19.89%). Numerous genes encoding transposase (Supplementary Table 1) were also observed, indicating that the genetic plasticity of the strain might be shaped by intragenomic rearrangements. It was proposed that transpositions play a crucial role in genomic rearrangements and are involved in gene regulation and adaptation processes that determine the directions of microevolutionary processes in cyanobacteria (Mikheeva et al., 2013).

The A183 chromosome harbored around 190 transporter-related genes (Supplementary Table 1). Among these transporters, ABC transporters accounted for the majority, and distinct bias was found in many genes, such as P-type ATPase transporter for copper, which only has two copies. Functionally, these transporters have been predicted as Na^+^/H^+^, iron, phosphate, amino acid, bicarbonate, CO_2_ transporters, etc.

### Phylogeny of 16S rRNA

**FIGURE 3:**
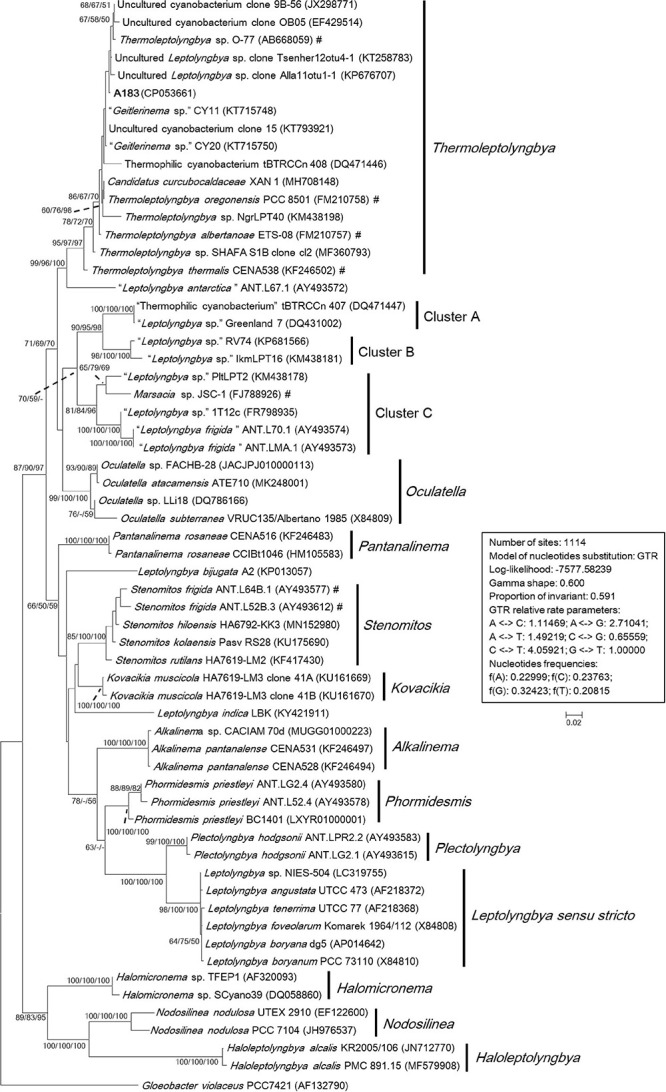
Maximum-likelihood phylogenetic tree of 16S rRNA gene sequences. The numbers at nodes refers to the support values of ML, MP, and NJ analysis, respectively. Only bootstrap values > 50% (1000 non-parametric replications) are indicated at nodes. Genera or cluster are indicated by vertical bars. Scale bar = 2% substitutions per site. Strains in quotation marks have uncertain genus names. Strains marked with # have been recently reclassified or proposed to be reclassified.

To ascertain the taxonomic position of strain A183, a ML phylogenetic tree was reconstructed based on 16S rRNA gene sequences of the 58 cyanobacterial strains. The ML tree (Figure 3) resolved 13 well-defined clades of isolates corresponding to previously described genera: *Alkalinema* (Vaz et al., 2015), *Halomicronema* (Abed et al., 2002), *Haloleptolyngbya* (Dadheech et al., 2012), *Kovacikia* (Miscoe et al., 2016), *Leptolyngbya sensu stricto* (Taton et al., 2010), *Nodosilinea* (Perkerson et al., 2011), *Oculatella* (Zammit et al., 2012), *Pantanalinema* (Vaz et al., 2015), *Phormidesmis* (Komárek et al., 2009), *Plectolyngbya* (Taton et al., 2011), *Stenomitos* (Miscoe et al., 2016), *Thermoleptolyngbya* (Sciuto and Moro, 2016), and *Gloeobacter* as an outgroup of the tree. Strain A183 investigated in this study closely clustered with 15 strains affiliated to genus *Thermoleptolyngbya*. Three clusters were not assigned to the previously described taxa and were marked as cluster A–C, respectively. In addition, the sequences of “*L. antarctica*” ANT.L67.1 and *L. indica* LKB did not collocate with any cluster and were placed in separate branches.

The sequence identities of 16S rRNA gene were calculated between A183 and other strains phylogenetically grouped into *Thermoleptolyngbya* clade (Supplementary Table 2). The A183 strain showed sequence identities ranging from 97.11 to 99.46%, compared to the other strains assigned to *Thermoleptolyngbya* clade. Notably, numerous strains within *Thermoleptolyngbya* clade were labeled by uncertain genus names (Figure 3), while the sequence identities of 16S rRNA gene (Supplementary Table 2) strongly indicated that these strains are members of the genus *Thermoleptolyngbya*, according to the recommended threshold for bacterial species (98–99%) or genera (94.5–95%) demarcation (Rodriguez-R et al., 2018). Moreover, these *Thermoleptolyngbya* strains originated from thermal environments worldwide (Supplementary Table 2; Lacap et al., 2007; Oren et al., 2008; Peng et al., 2013; Nakamori et al., 2014; Gaisin et al., 2015; Bravakos et al., 2016; Sciuto and Moro, 2016; Heidari et al., 2018; Tang et al., 2018b), except for strain XAN 1 and CENA538. This is consistent with the general knowledge that organisms belonging to *Thermoleptolyngbya* appear to have thermal origins (Sciuto and Moro, 2016). Information on XAN 1 is sparse, and perhaps it was an inhabitant of hydrothermal spring based on the submission title on NCBI. CENA538 isolated from saline-alkaline Lake during the Brazilian dry season was subjected to desiccation periods, and the temperature and high salinity of the sampling site could be considered as a thermal environment (Andreote et al., 2014).

The results of phylogenetic reconstruction (Figure 3) and sequence identity (Supplementary Table 2) indicated that 16S rRNA gene might not effectively differentiate the species-level relationship of strains belonging to *Thermoleptolyngbya*. A verified example was the establishment of two different *Thermoleptolyngbya* species (*T. oregonensis* and *T. albertanoae*) (Sciuto and Moro, 2016), whereas the phylogenetic relationship (Figure 3) and sequence identity (99.17%) of the two strains were susceptible to reach an erroneous conclusion on species differentiation. Therefore, a polyphasic approach encompassing morphological, molecular, and phylogenetic analysis of more genomic loci is inordinately crucial for accurate species identification within *Thermoleptolyngbya*.

### Phylogeny of 16S-23S ITS

**FIGURE 4:**
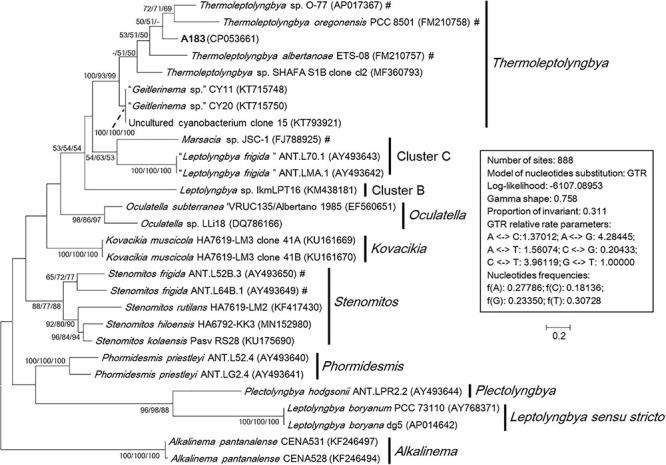
Maximum-likelihood phylogenetic tree of 16S-23S ITS sequences. The numbers at nodes refers to the support values of ML, MP, and NJ analysis, respectively. Only bootstrap values > 50% (1000 non-parametric replications) are indicated at nodes. Genera or cluster are indicated by vertical bars. Scale bar = 20% substitutions per site. Strains in “quotation marks” have uncertain genus names. Strains marked with # have been recently reclassified or proposed to be reclassified.

The phylogenetic reconstruction based on full-length 16S-23S ITS sequences (Figure 4) was consistent with the phylogeny of the 16S rRNA gene, although fewer sequences were included in the analysis. The *Alkalinema* clade was rooted as an outgroup. Strains ascribable to *Thermoleptolyngbya* were placed into a well-supported clade and showed evident genetic divergence as revealed by branch length (Figure 4), indicating that there might be six species within genus *Thermoleptolyngbya*. The 16S-23S ITS tree also detected clades corresponding to previously described genera supported by robust bootstrap values, namely *Alkalinema*, *Kovacikia*, *Leptolyngbya sensu stricto*, *Oculatella*, *Phormidesmis*, *Plectolyngbya*, and *Stenomitos*.

There are two primary reasons attributed to fewer sequences included in the 16S-23S ITS phylogenetic analysis than that in 16S rRNA analysis. Firstly, the 16S-23S ITS sequences are unavailable for many strains. In this study, only half of *Thermoleptolyngbya* strains had both of their 16S rRNA and 16S-23S ITS sequences determined, hindering further comprehensive taxonomic recognition. Secondly, more importantly, the 16S-23S ITS marker is highly variable and is difficult to be aligned precisely if distantly related taxa were included in the database, affecting the outcome of phylogenetic reconstructions. It has been suggested that reliable phylogenies of 16S-23S ITS can be achieved by limiting the analysis to highly related taxa and with the support of secondary structure analysis (Johansen et al., 2011, 2014). However, to ascertain the species identity, a more accurate analysis at molecular level is required, utilizing MLSA and eventually whole genome sequence comparison.

The 16S-23S ITS marker contains highly variable regions and highly conserved domains. The sequence identities are distinct when using full-length ITS or regions/domains individually, probably bringing about misleading information without secondary structure domain analysis. This speculation has been manifested by previous reports (Johansen et al., 2011; Sciuto and Moro, 2016). Additionally, the hyper-variable region of 16S-23S ITS marker is nearly neglected in the phylogenetic analysis since gapped positions are excluded. Those regions, however, may be informative at the species level. Therefore, secondary structure domain analysis of 16S-23S ITS is essential as a complement to ultimate taxonomy determination and is beneficial for better resolving the taxonomic status of problematic strains with inconsistent phylogenetic inferences between 16S rRNA and 16S-23S ITS.

### Secondary Structures of 16S-23S ITS

**FIGURE 5:**
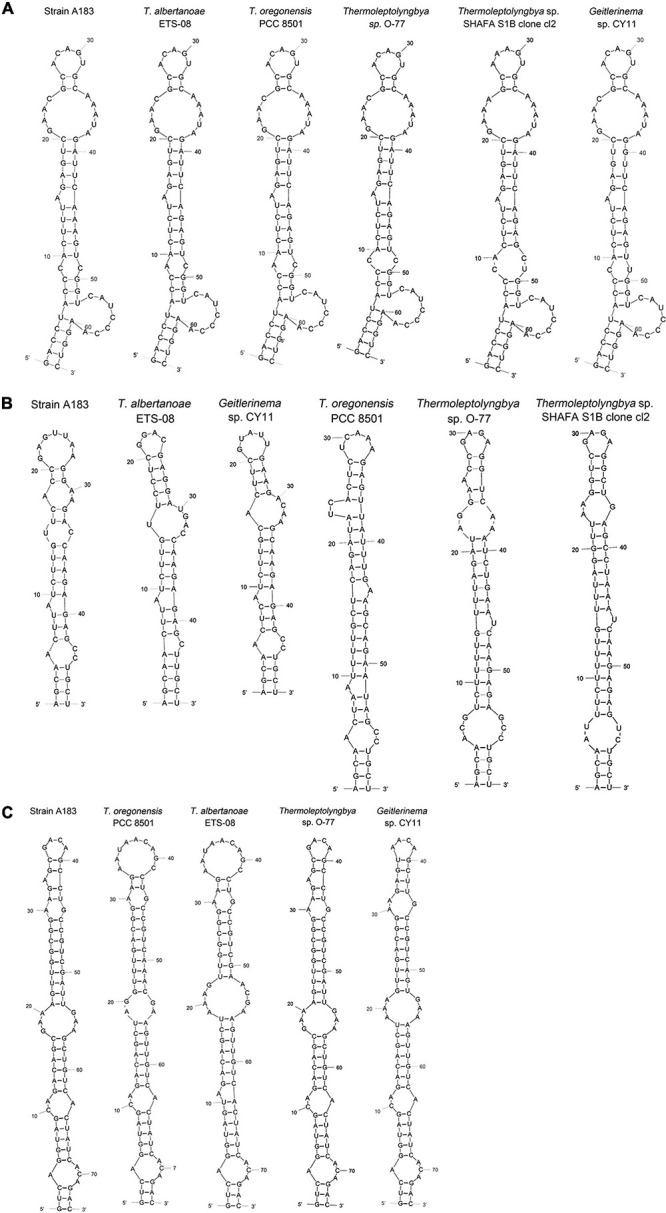
Predicted secondary structures of D1-D1’ helix **(A)**, boxB **(B)**, and V3 helix **(C)** of 16S-23S ITS of *Thermoleptolyngbya* strains.

Hypothetical secondary structures of domains within ITS were estimated for strain A183 and representative strains from genus *Thermoleptolyngbya* in the 16S-23S ITS tree (Figure 5). Excluding two highly conserved tRNAs from full-length ITS sequences, the length of the remaining ITS sequences varied greatly from 297 to 535 bp (Table 4). The remaining ITS sequence of A183 strain was the longest among *Thermoleptolyngbya* strains, 535 bp in length. Identical sequences were observed in conserved domains D3 (GGTTC), boxA (GAACCTTGAAAA), and D4 (CTGCATA) among all *Thermoleptolyngbya* strains. Conserved domain D2 showed four sequence types with slight nucleotide difference, namely CTTCCAAACTAT in A183, O-77 and SHAFA S1B clone cl2, CTTCCAAGCTAG in ETS-08, CTTCCAAACTGT in CY11, and TTTCCAAACTAT in PCC 8501.

**TABLE 4:** The length (bp) summary of regions within 16S-23S ITS of strains studied.

**Strain**	**ITS length (tRNA removed)**	**D1-D1’ helix**	**D2**	**D3**	**boxA**	**D4**	**V2 helix**	**boxB helix**	**V3 helix**
A183	535	64	12	5	12	7	218	48	74
*Thermoleptolyngbya albertanoae* ETS-08	441	64	12	5	12	7	152	47	74
*Thermoleptolyngbya oregonensis* PCC 8501	344	64	12	5	12	7	75	60	74
*Thermoleptolyngbya* sp. O-77	486	64	12	5	12	7	211	60	74
*Thermoleptolyngbya* sp. SHAFA S1B clone cl2	358	64	12	5	12	7	39	60	NA
*Geitlerinema* sp. CY11	297	64	12	5	12	7	11	48	74

*NA, not available.*

The inferred D1-D1’ helices were drawn in Figure 5A. All helices exhibited the same length and nearly identical structures. However, nucleotide differences of D1-D1’ primary structures were found among strains. From basal stem (GACCU-AGGUC) onward, all the helices were structured by seven residue asymmetrical loops followed by three residue stems, two residue symmetrical loops followed by five residue stems, and one base left bulge followed by five residue stems, nine residue asymmetrical loops, two residue stems, and five residue asymmetrical loops. The only outlier of this structure is SHAFA S1B clone cl2 where seven residue asymmetrical loops and three residue stems are followed by four residue stems and four residue symmetrical loops; all the remaining parts of the structure were unchanged.

Hypothetical V2 helices were tremendously distinct among *Thermoleptolyngbya* strains (Supplementary Figure 3), and no common basal structure was found. The highly variable helices were attributed to the divergent primary structures of V2 regions. The longest V2 helix was found to be 218 residues in strain A183, followed by 211 residues in strain O-77, 152 residues in ETS-08, and 75 residues in PCC 8501, respectively (Table 4). The V2 helices of the remaining two strains were only 11 and 39 residues in length (Table 4).

The depicted boxB helices (Figure 5B) indicated that a basal stem structure (AGCA-UGCU) was shared by all *Thermoleptolyngbya* strains. Although A183 had similar residue length to ETS-08 and CY11 (Table 4), the boxB helix structure of A183 was clearly distinct from that of the two strains. The boxB helix of A183 was mainly composed of a stem orderly fragmented by three residue asymmetrical loops, single base left bulge, two residue symmetrical loops, three residue asymmetrical loops, and terminated with seven residue hairpin loops. The boxB helices of the other *Thermoleptolyngbya* strains (Figure 5B) all terminated with hairpin loops variable in residues sequence and length, while the main stem structure of boxB helices considerably varied among strains in single base right bulge (in EST-08, PCC 8501, O-77, and SHAFA S1B clone cl2), single base left bulge (in EST-08, PCC 8501, O-77, and CY11), two base left bulge (in PCC 8501), asymmetrical loop (in EST-08, PCC 8501, SHAFA S1B clone cl2, and CY11), and symmetrical loop (in O-77).

The V3 helices shared a basal stem structure (GUC-GAC) among all *Thermoleptolyngbya* strains (Figure 5C). The V3 helix of A183 comprised two asymmetrical loops, two symmetrical loops, single base left bulge, fragmented stems, and terminated with four residue hairpin loops. Although all the V3 helices showed the same helix length (Table 4), the structures (Figure 5C) differed from each other in terms of bulge, loop, and stem. Unfortunately, V3 helix of SHAFA S1B clone cl2 was not inferred due to incomplete sequences in this region.

In summary, the secondary structures of V2, boxB, and V3 undoubtedly differentiate A183 from the other *Thermoleptolyngbya* strains, whilst the structure of the D1-D1’ domain remains conserved. The result of the 16S-23S ITS secondary structure analysis is in agreement with the phylogenetic reconstructions of 16S-23S ITS, confirming the verification of the A183 strain as a new species of *Thermoleptolyngbya*. The secondary structure analysis of V2, boxB, and V3 helices appeared to be effective for species-level identification. Although the V2 helix was the most variable, it was the least taxonomic-informative in light of its high variability and absence in some cyanobacterial strains (Iteman et al., 2000; Sciuto and Moro, 2016). Although it was reported that D1-D1’ helix, compared to boxB and V3 helix, is more important for interspecies discrimination within a given genus (Perkerson et al., 2011; Vieira Vaz et al., 2015), it turned out to be not that effective in the case of *Thermoleptolyngbya*. The utilization of boxB and V3 for species distinction within *Thermoleptolyngbya* has been verified by the successful differentiation of *T. albertanoae* and *T. oregonensis* (Sciuto and Moro, 2016).

Interestingly, phylogenetic analysis and secondary structure analysis of 16S-23S ITS both indicated that *Thermoleptolyngbya* strains listed in Table 4 are probably different species to each other within the genus *Thermoleptolyngbya*, even though the phylogeny (Figure 2) and sequence identity (Supplementary Table 2) of 16S rRNA did not initially reveal such differentiation. Undoubtedly, detailed information regarding morphology and DNA sequence of more loci or complete genome would be essential for the taxonomic revision of SHAFA S1B clone cl2 and CY11within genus *Thermoleptolyngbya*.

### Comparative Genome Analyses

**FIGURE 6:**
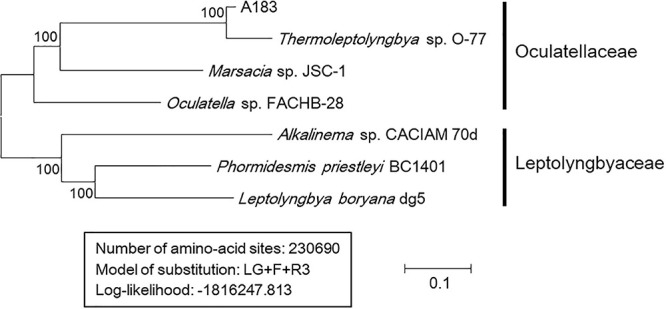
Maximum-likelihood phylogenomic tree of concatenated protein alignment of 751 single-copy genes shared by all genomes. Bootstrap values (1000 replications) are indicated at nodes. Scale bar = 10% substitutions per site.

As shown in Table 5, results of genome-wide ANI and AAI conformed to the suggested values for species (ANI > 96%, AAI ≥ 95%) and genus (ANI < 83%, AAI ≤ 70%) delimitation (Walter et al., 2017; Jain and Rodriguez, 2018), again confirming the taxonomy delineation of strain A183 as a novel species of *Thermoleptolyngbya*. Moreover, the concatenated alignment of 751 single-copy genes from the complete genomes of related strains produced an ML genomic tree with 100% bootstrap support (Figure 6), the topology of which was consistent with that of 16S rRNA and ITS. All the results verified the conclusion that strain A183 was a novel species of *Thermoleptolyngbya*. Unfortunately, the unavailability of genomes led to the failure of a comprehensive snapshot of divergences in genomes among all strains related to A183. To compensate for this shortcoming, partial data extracted from the metagenomic bins that show resemblance to *Thermoleptolyngbya* have been also analyzed with the combination of MLSA (Supplementary Figure 4), patristic distance (Supplementary Table 3), and *in silico* DNA-DNA hybridization (Supplementary Table 4) approaches. Provided analyses further reinforce the claim that the newly isolated strain is a novel species within the *Thermoleptolyngbya* genus. The A183 strain clearly separates from previously described O-77 and PCC 8501 strains. Interestingly, two recently deposited metagenomic bins (C42_A2020 and M55_K2018) obtained from the hot springs of Cahuelmo, Chile (temperature 42°C, pH 9.2) and Chhattisgarh, India (temperature 55°C, pH 7.9) show sequences of closer resemblance to A183 than either O-77 or PCC 8501. The lack of fully sequenced genomes and some key phylogenetic markers such as 16S or ITS as well as morphological data makes this analysis somehow incomplete and prevents them from being unequivocally described as members of *Thermoleptolyngbya*. These results, however, support delineation of a new species around A183, and an implied global distribution of *Thermoleptolyngbya*, indicating also potentially novel, closely related filamentous cyanobacteria.

**TABLE 5:** Summary of ANI (Average Nucleotide Identity) and AAI (Average Amino acid Identity) between cyanobacterial genomes studied.

**Strain**	**A183**	**O-77**	**70d**	**dg5**	**JSC-1**	**FACHB-28**	**BC1401**
A183	100	93.59	59.18	60.24	64.54	64.59	61.36
*Thermoleptolyngbya* sp. O-77	89.97	100	58.64	59.75	64.12	63.99	60.95
*Alkalinema* sp. CACIAM 70d	74.88	75.95	100	61.17	59.35	58.18	62.17
*Leptolyngbya boryana* dg5	75.80	76.35	76.91	100	60.51	60.58	66.71
*Marsacia* sp. JSC-1	75.55	74.88	79.21	75.05	100	64.96	61.34
*Oculatella* sp. FACHB-28	74.12	74.00	74.83	76.25	75.06	100	62.73
*Phormidesmis priestleyi* BC1401	75.25	74.57	74.39	75.05	73.85	74.52	100

*The numbers above and below the diagonal indicate the AAI and ANI values (%), respectively.*

To have an in-depth understanding of the A183 strain functioning in its ecosystem and to clarify the genetic features of the strain, a comparative genome analysis was performed between the A183 strain and its relative *Thermoleptolyngbya* (O-77). Generally, the two strains shared a similar genome size, GC content, and the number of rRNA operons and tRNAs, but a significant difference in CDS number (Table 3). The subsystem category distribution was also almost identical to both strains (Supplementary Figure 5). Analogously, gene ontology (GO) analysis of all CDS showed a similar distribution of functional categories in both strains (Supplementary Figure 2).

An ortholog table (Supplementary Table 5) was constructed based on all-against-all BLASTP alignment. As indicated in Figure 2B, strain A183 shared 3,974 gene-encoding homologous sequences with strain O-77, accounting for 76.9% annotated genes of the A183 genome. Unique genes were also found in each strain, namely 1,192 genes in A183 and 1,717 genes in O-77 (Figure 2B). Further, GO analysis of the unique genes showed that these genes were distributed in a wide range of functional category (Supplementary Figure 6).

No intact prophage region was detected in either strain. Three and 11 CRISPR arrays were identified in the genomes of A183 and O-77, respectively (Supplementary Table 6). A higher number of spacers (597) was observed in the O-77 genome, approximately 3.5 times higher than that of A183. All direct repeats of the CRISPR arrays were 35 bp long in the A183 genome, while there were two kinds of length (34 and 35 bp long) in the O-77 genome. The A183 had only one CRISPR array with adjacent Cas genes. Although O-77 had two CRISPR arrays with Cas genes, one of them did not have a full set of genes encoding the Cas system and was not assigned to a specific CRISPR-Cas system. The type III-D CRISPR-Cas system in both strains might confer resistance to foreign mobile genetic elements from bacteriophage or viruses (Makarova et al., 2011).

### Thermotolerance

A183 was originally isolated from the hot spring with a temperature of 40.8°C (Tang et al., 2018b) and is capable of growing at a maximum of 50°C (Tang et al., 2018a). A survival strategy must be well-prepared for A183 to survive in these thermal environments. It is known that heat shock proteins (Hsps) are essential for maintaining and restoring protein homeostasis. In the A183 genome, dozens of genes were identified to encode heat shock proteins (Hsps), including the Hsp100, Hsp90, Hsp70, and Hsp60 family as well as small Hsps.

Hsps belonging to Hsp100 family may favor the refolding, disaggregation, and removal of heat-damaged proteins. For instance, ATP-dependent protease *clpX*, associated with *clpP*, promoted disassembly and degradation of heat-aggregated substrates (e.g., *ftsZ*) (Labreck et al., 2017). Homologs of the *clp* family were found in the A183 genome: *clpB*, -*C*, -*P*, -*S*, -*X* (TS0414, TS0934, TS1039, TS1204-1206, TS1331, TS1444, TS1861-1862, TS2363, TS3986, and TS4547). An identical composition and distribution of *clp* genes were found in the O-77 genome (Supplementary Table 1).

There was only one copy of the *htpG* gene (TS0820) in A183, and so was O-77 (Supplementary Table 1). The *htpG* protein of the Hsp90 family was suggested to be more of a general stress protein in that it played a role in several abiotic stresses (Hossain and Nakamoto, 2002, 2003). Particularly, the *htpG* primarily protected the photosynthetic machinery from heat stress in cyanobacteria (Sato et al., 2010).

Proteins of Hsp70 family appeared to be prosperous in the A183 genome, mainly including *dnaK* (TS1830, TS2881, TS4129, and TS4674) and *dnaJ* (TS1379, TS2355, TS2878, TS4052, TS4637, TS4910, and TS5059). Multiple copies of *dnaK* and *dnaJ* genes have also been reported in other cyanobacteria (Rajaram et al., 2014). Nevertheless, it was suggested that *dnaK* and *dnaJ* proteins might function differently, and only some of them contributed to thermotolerance (Schneider, 2010; Duppre et al., 2011). Moreover, the gene *grpE* (TS2882) was found, the protein encoded by which might act as a cofactor of Hsp70 family and participate actively in response to heat shock by preventing the aggregation of stress-denatured proteins (Schneider, 2011). The homologs of Hsp70 family in O-77 showed a high similarity to that in A183 (Supplementary Table 1).

Interestingly, two distinct *groEL* genes (TS0663, TS2946) of the Hsp60 family, also referred to as the *groE* chaperone machinery, were identified in the A183 genome. One of them (TS2946, *groEL*-1) formed *groESL* operon together with the small Hsp *groES* (TS2947). The *Gloeobacter* PCC 7421 genome contains two *groEL* genes, both of which have *groES* immediately upstream of each *groEL*. Therefore, it was speculated that one of the two *groESL* operons had lost its *groES* during the evolutionary cyanobacterial diversification. The *HrcA* repressor system (TS4519) was also found in the A183 genome, which may negatively regulate the expression of *groE* genes (Nakamoto et al., 2003). A similar composition of *groE* genes was observed in the O-77 genome.

In addition to the genes mentioned above, genes encoding small Hsps and proteases were also identified in the A183 genome (Supplementary Table 1) and might be involved in thermotolerance. For example, small *Hsp16* (TS0692), also referred to as *hspA*, may act as a chaperone and interact with dozens of proteins at high temperature, playing multiple roles ranging from protein folding to stabilization of thylakoid and periplasmic membranes (Basha et al., 2004); the *ftsH* protease (TS0606, TS1376, TS1741, TS1916, and TS2838) may be responsible for heat-induced degradation of photodamaged D1 protein by up-regulated expression of *ftsH* (Kamata et al., 2005). However, further, detailed investigations on the actual functions of these genes are necessary to elucidate the mechanisms of adaptation of A183 to high temperatures. Based on previous results concerning the thermophilic cyanobacteria from this region’s thermal springs it could be concluded that high temperatures are physiological to these strains and the putative thermotolerance genes are expressed constitutively (results not published). In order to fully elucidate the impact of these genes on strain thermostability, a loss of function study, such as targeted gene knock-out or silencing, will be needed. This in turn, requires development of the entire repertoire of genetic tools that is currently unavailable for *Thermoleptolyngbya* sp. and related strains.

### Signal Transduction

The two-component regulatory systems have been commonly observed in cyanobacteria and elucidated for the perception of environmental stress and the subsequent transduction of stress signals (Los et al., 2010). In the A183 genome, 36 and 31 genes were identified to encode histidine kinases and response regulators, respectively (Supplementary Table 1). The system composed of these genes is likely to play major roles in the core part of acclimation to changing environments. However, the scattered distribution of these genes in the A183 genome hindered the association of genes for histidine kinases with their respective cognate response regulators. This was in sharp contrast to cases in *E. coli* (Bourret and Silversmith, 2010) or *Bacillus subtilis* (Aguilar et al., 2001) that the genes for a single two-component system were organized into operons or located close one to another. Thus, investigation on sensors and their cognate regulators requires individual mutation on these genes. Similarly, the scattered genes encoding histidine kinases and response regulators were also found in the O-77 genome (Supplementary Table 1).

The serine/threonine protein kinases (Spks) had similar purposes as two-component systems for signal transduction. A total of 17 genes were identified in A183 genome to encode Spks (Supplementary Table 1). Unfortunately, the functions of only several Spks in *Synechocystis* have been characterized to date, such as *spkA* and *spkB* involved in the control of cell motility, and *spkE* involved in the regulation of nitrogen metabolism (Zhang et al., 2006). Although the Spks proteins were conserved among *Thermoleptolyngbya* A183 and O-77 strains as revealed by their high similarity of amino acid sequences (87.61–96.71%, Supplementary Table 5), the Spks proteins of *Thermoleptolyngbya* strains were quite divergent from those of other cyanobacterial strains. These putative Spks proteins in *Thermoleptolyngbya* are required to be more carefully investigated in the future.

Different environmental conditions or developmental signals often cause major changes in transcription pattern by inducing a swap of sigma factors in the RNA polymerase holoenzyme (Los et al., 2010). The A183 genome inhabited seven genes encoding Group 2 sigma factor *sigD* (TS0188, TS0493, TS1286, TS1338, TS2057, TS3542, and TS4081) and two genes encoding Group 3 sigma factor *sigF* (TS1335 and TS3418). The *sigD* is the only *sig* gene that produced moderate amounts of transcripts in the dark and was not affected by any of the stress treatments (Tuominen et al., 2003), suggesting its crucial role in transcription regulation particularly in adverse conditions. The exact function of *sigD* in cyanobacteria remains to be clarified.

In addition, seven genes encoding GGDEF/EAL domain proteins were observed in the A183 genome (Supplementary Table 1). It was reported that GGDEF/EAL domain proteins function as diguanylate cyclase/phosphodiesterase that synthesizes/degrades cyclic di-GMP and participate in a cyclic-di-GMP signaling pathway that may regulate biofilm formation, motility, virulence, and cell cycle (Agostoni et al., 2013). A recent study showed that GGDEF/EAL domains were also involved in blue-light-induced cell aggregation in *Thermosynechococcus* BP-1 and NIES-2134 (Enomoto et al., 2015). Interestingly, *Thermosynechococcus* genomes have 9–13 genes encoding GGDEF/EAL domains, while hot-spring *Synechococcus* genomes (strain JA-3-3-Ab and JA-2-3Ba) only have four genes (Cheng et al., 2020). These data implied that the complexity of cyclic-di-GMP signaling pathways appeared to be distinct among thermotolerant strains.

### Carbon Assimilation

Cyanobacteria in hot springs have to deal with many environmental stresses, one of which is low CO_2_ solubility at high temperatures. The CO_2_-concentrating-mechanism cyanobacteria have evolved into can partially alleviate this problem by actively transporting and accumulating inorganic carbon (Ci: CO_2_ and HCO_3_^–^) for the sake of a satisfactory rate of CO_2_ fixation under carbon-limiting concentrations (Price et al., 2008). The uptake of gaseous CO_2_ systems in cyanobacteria relied on NADPH dehydrogenase (NDH-1) complexes (Price et al., 2008). In the A183 genome, there were 19 NDH-1 genes encoding *ndhA*, *ndhB*, *ndhC*, *ndhE*, *ndhF*, *ndhG*, *ndhH*, *ndhI*, *ndhJ*, *ndhK*, *ndhL*, and *ndhM*, respectively (Supplementary Table 1). Except for *ndhE* and *ndhF*, the other NDH-1 genes were present in one copy. Meanwhile, several genes clustered together (*ndhE*-*ndhG*-*ndhI*-*ndhA*, TS1642-1645; *ndhJ*-*ndhK*-*ndhC*, TS2756-2758; *ndhF*-*ndhE*, TS4963-4964), while *ndhE* and *ndhF* were composed of clusters coupled with low-affinity CO_2_ hydration proteins, namely *ndhF*-*ndhE*-*cphY* (TS4168-4170) and *ndhF*-*ndhE*-*cphX* (TS4275-4277). These gene clusters probably contribute to constitutively expressed NDH-1 complex involved in low-affinity CO_2_ uptake (Shibata et al., 2001). Similar clusters were also found in the O-77 genome.

Our previous study showed that *Thermoleptolyngbya* A183 survived at the concentration exceeding 0.5 M NaHCO_3_ (Tang et al., 2018a), suggesting that the A183 strain can assimilate bicarbonate and convert it to CO_2_ for photosynthesis. This was further evidenced by the HCO_3_^–^ uptake systems, as suggested by the genome analysis. First, a homolog (TS0333) of a low-affinity, high flux, Na^+^-dependent bicarbonate transporter (*bicA*) was found in the A183 genome. Second, the A183 genome also harbored a homolog (TS2219) of *sbtA*, an inducible, high-affinity Na^+^-dependent bicarbonate transporter (Shibata et al., 2002). The transporter shows 69.3% sequence identity to *sbtA* of other thermophilic cyanobacterium *Thermosynechococcus lividus* PCC6715 (Liang et al., 2019). The different bicarbonate uptake systems might be flexibly utilized by A183 to meet the conditional demand for carbon gain. Interestingly, the *BCT1* bicarbonate transporter typical for many other cyanobacteria was not detected in the A183 genome, suggesting a difference regarding a bicarbonate uptake mechanism from *Thermosynechococcus* BP-1 that lacks *sbtA* entirely but is equipped with *BCT1* (Price et al., 2008). In addition, three ABC-type bicarbonate transporters were observed in the A183 genome. The O-77 genome also possessed genes encoding *BicA* and *sbtA* and showed high protein similarities with A183 (94.99 and 95.56%, respectively), but the number of genes encoding an ABC-type bicarbonate transporter was higher than that of A183 (9 vs. 3). These results are consistent with the physiological characterization of the strain (Table 2).

### Nitrogen Assimilation

In the A183 genome, eight genes encoding nitrogenases were identified, including *nifB*, *nifR*, *nifH*, *nifO*, *nifW*, and *nifX* (Supplementary Table 1). These genes encode proteins required for catalytic activity, Fe–Mo cofactor biosynthesis, and maturation and stability of the nitrogenase protein complex (Steunou et al., 2008). O-77 exhibited similar gene components regarding nitrogenases. These results suggested that both strains are nitrogen-fixing non-heterocystous cyanobacteria. N_2_ fixation is an energetically expensive metabolic reaction catalyzed by nitrogenase, which is inhibited by O_2_ generated during photosynthesis (Steunou et al., 2008). Therefore, *Thermoleptolyngbya* A183 also exhibits alternative strategies in light of an economy of nitrogen assimilation.

Specific transporters are essential for organisms to concentrate ambient nitrogen sources within cells to survive in oligotrophic aquatic environments (Esteves-Ferreira et al., 2018). In the A183 genome, the ABC-type nitrate transport system (*nrtABCD*, TS4258-4261) was detected and formed an operon with two essential nitrogen-related genes located at both sides of *nrtABCD*, encoding ferredoxin-nitrite reductase (*nirA*, TS4257) and ferredoxin-nitrate reductase (*narB*, TS4262), respectively. This result was consistent with many freshwater cyanobacterial strains (Maeda et al., 2015). Furthermore, a complete gene set (*urtABCDE*) of ABC-type urea transport system and seven genes encoding urease (*ureA* – *ureG*) was observed (Supplementary Table 1), suggesting the ability of A183 to import and utilize urea as a nitrogen source. In addition, two genes were identified as ammonium transporter (TS132, TS5152). The O-77 genome showed similar components of nitrogen-related transporters. The above results reinforced the importance of these transporters for cyanobacterial growth in oligotrophic environments and also implied that A183 can depend on multiple forms of nitrogen sources. The ability of both strains to fix molecular nitrogen and utilize urea has been confirmed experimentally (Table 2).

### Sulfur Assimilation

The A183 strain was isolated from a hot spring abundant in sulfur (Tang et al., 2018b). The analysis of the A183 genome reveals three transporter systems involved in sulfate uptake. First, the sulfate-thiosulfate permease (*sulT*), belonging to the ABC-type transporter, comprised four subunits encoded by the *cysPTW* operon (TS1272-1274) and *cysA* (TS4980) that was located far away from the operon. The O-77 genome showed a different operon in terms of *cysPTW* but a similar distribution of *cysA* gene. The organization of the *cysPTW* operon in the *Thermoleptolyngbya* strains was different from that of *Thermosynechococcus* strains, such as BP-1 and PKUAC-SCTE542, resulting from the replacement of *cysP* by *sbpA* (Liang et al., 2019). The second sulfate permease in the A183 genome was *sulP* (TS1400), encoded by a single polypeptide and functioned as inorganic anion uptake carriers or anion:anion exchange transporters (Aguilar-Barajas et al., 2011). No homolog of *sulP* was identified in the O-77 genome. Besides, sulfate can also be transported by the high-affinity ModABC molybdate transport system (Aguilar-Barajas et al., 2011), and the ModABC transporter in the A183 genome was encoded by the *modABC* operon (TS2382-2384). An analogous ModABC transporter was also found in the O-77 genome. The predominant pathway and substrate specificity need to be experimentally clarified in future research. Physiological studies confirmed the strain’s ability of growth in the presence of sulfates (Table 2).

## Conclusion

The polyphasic approach used in this study, including phylogenetic, phylogenomic, ultrastructural, physiological, and morphological surveys, came up with a proposal of a new species, *Thermoleptolyngbya sichuanensis*, and the delineation of this new taxon around the representative strain A183. Although basic phylogenetic analysis and sequence identities of 16S rRNA showed high similarity among *Thermoleptolyngbya* strains, more advanced genome-based approaches strongly confirmed the delineation of a new species of *Thermoleptolyngbya*. Analysis of metagenome-assembled genomes revealed that the proposed species is not endemic but present in hot springs globally. Meanwhile, comparative genome analysis revealed distinct genome structures of *Thermoleptolyngbya* strains. Moreover, genes related to thermotolerance, signal transduction, and carbon/nitrogen/sulfur assimilation were thoroughly analyzed and partially verified experimentally for illustrating the ability of this strain to adapt to inhospitable niches in hot springs.

### Taxonomic Treatment and Description of *Thermoleptolyngbya sichuanensis* Daroch, Tang, and Shah et al. sp. nov.

The classification system that was applied was based on Komarek et al. (2014).

Taxon description in accordance with the prescriptions of the International Code of Nomenclature for Algae, Fungi, and Plants (Shenzhen code) (Turland et al., 2018).

Phylum: Cyanobacteria

Order: Synechococcales

Family: Leptolyngbyaceae

*Description:* The cell morphology of the A183 strain indicated by light microscopy revealed straight, wavy, and occasionally bent trichomes (Figure 1A). The SEM and TEM (Figures 1B–D) showed that trichomes of strain A183 were unbranched and composed of 80–120 elongated barrel-shaped cells, 1.30–1.60 μm in length and 1.05–1.10 μm in width. Constrictions were observed at the cross-walls of cells (Figures 1B–D). Individual cells of the filaments were divided by centripetal invagination of the cell wall (Figure 1E). Intracellular connections between vegetative cells were not observed (Figure 1D). The TEM analysis also exhibited that the three to five thylakoid layers were located in parallel at the inner periphery of cells (Figures 1C,D) and can be described as parietal according to recent classification (Mareš et al., 2019). Sheath, septum, phycobilisome, carboxysomes, cyanophycin granule, and polyphosphate bodies were present in the cytoplasm (Figures 1C–F), and small lipid droplets were also observed (Figure 1C).

*Type strain:* is A183 (= FACHB-2491).

*Etymology:* Species epithet derives from the name of collection site.

*Type locality:* Thermal springs in Ganzi Prefecture of Sichuan Province, China.

Ecology of type locality: the sample occurred as macroscopic green mat attached to the sinter around the pond with a small amount of mucilage around the entire mat. Sample collection was done in 12.05.2016 with the humidity being close to 71%. Temperature at the time of collection was 15°C and the light intensity was around 1000 lux. The pH of the spring was 6.32 and concentration of total dissolved solids was 447 mmol L^–1^

*Habitat:* thermal springs in Ganzi Prefecture of Sichuan Province, China (30°05’14” N, 101°56’55” E) *Thermoleptolyngbya* species exhibiting peak NaHCO_3_ tolerance as high as 1 M, and 0.5 M during prolonged cultivation. The strain can utilize urea up to a concentration of 3 mM and withstand 10 mM Na_2_SO_4_, but not an equivalent concentration of Na_2_HSO_3_. The strain is diazotrophic and exhibits experimentally verified nitrogenase activity with acetylene reduction assay (Table 2 and Supplementary Figure 1).

*Holotype here designated:* the culture of *Thermoleptolyngbya sichuanensis* was initially denoted and deposited in Peking University Algae Collection as PKUAC-SCTA183 has also been deposited in the Freshwater Algae Culture Collection at the Institute of Hydrobiology (FACHB-collection) with accession number FACHB-2491 as *Thermoleptolyngbya* species after identification and authentication on the basis of the full-length sequencing of the 16S rRNA gene along with folding of the secondary structures of the 16S–23S ITS region. After proper identification and authentication, the culture is being maintained in the FACHB under the accession number FACHB-2491.

## Data Availability Statement

The datasets presented in this study can be found in online repositories. The names of the repository/repositories and accession number(s) can be found below: https://www.ncbi.nlm.nih.gov/genbank/, CP053661.

## Author Contributions

JT: conceptualization, methodology, validation, formal analysis, investigation, data curation, writing-original draft, writing-review and editing, visualization, supervision, project administration, and funding acquisition. LL and ML: formal analysis, investigation, data curation, and writing-original draft. LD and MMW: methodology, software, and data curation. MS: methodology, validation, and writing-original draft. MW: methodology and writing-review and editing. KW: conceptualization, methodology, and writing-review and editing. MD: conceptualization, methodology, resources, data curation, writing-original draft, writing-review and editing, supervision, project administration, and funding acquisition. All authors contributed to the article and approved the submitted version.

## Conflict of Interest

The authors declare that the research was conducted in the absence of any commercial or financial relationships that could be construed as a potential conflict of interest.

## Publisher’s Note

All claims expressed in this article are solely those of the authors and do not necessarily represent those of their affiliated organizations, or those of the publisher, the editors and the reviewers. Any product that may be evaluated in this article, or claim that may be made by its manufacturer, is not guaranteed or endorsed by the publisher.

## Footnotes

1
http://enve-omics.ce.gatech.edu/ani/
