# Supplementary material for: Description, Taxonomy, and Comparative Genomics of a Novel species, Thermoleptolyngbya sichuanensis sp. nov., Isolated From Hot Springs of Ganzi, Sichuan, China
Source: Front Microbiol. 2021 Sep 10;12:696102. doi: 10.3389/fmicb.2021.696102 (PMC8461337; doi:10.3389/fmicb.2021.696102)
Supplement: Supplementary file 2 [file Table_2.DOCX]

**Table S2** The sequence identities of 16S rRNA gene between strain A183 and other *Thermoleptolyngbya* strains. Number in brackets indicated the pairwise alignment length. Strains are sorted by the order of identity from high to low. A dash refers to data unavailable. Strains in “quotation marks” have uncertain genus name.

| Strain | Sequence identity with A183 (%) | Isolation source | Reference |
| --- | --- | --- | --- |
| A183 | 100 (1494) | Hot spring in Erdaoqiao, Ganzi Prefecture, Sichuan, China | Tang et al., 2018b |
| *Thermoleptolyngbya* sp. O-77 | 99.46 (1489) | Hot spring in Aso-Kuju National Park, Kumamoto, Japan | Nakamori et al., 2014 |
| “*Geitlerinema* sp. ” CY11 | 99.40 (1172) | Afyonkarahisar hot spring, Turkey | NA |
| Uncultured cyanobacterium clone 15 | 99.32 (1170) | Afyonkarahisar hot spring, Turkey | NA |
| Uncultured “*Leptolyngbya* sp.” clone Tsenher12otu4-1 | 99.24 (1452) | Tsenher hot spring, Mongolia | NA |
| “*Candidatus curcubocaldaceae*” XAN 1 | 99.13 (1493) | NA | NA |
| “*Geitlerinema* sp.” CY20 | 99.06 (1175) | Afyonkarahisar hot spring, Turkey | NA |
| *Thermoleptolyngbya oregonensis* PCC 8501 | 98.89 (1445) | Hunter's hot spring, Oregon, USA | Sciuto and Moro, 2016 |
| Uncultured cyanobacterium clone OB05 | 98.63 (1454) | Wonder Lake geothermal location, Luzon Island, Philippines | Lacap et al., 2007 |
| *Thermoleptolyngbya* sp. SHAFA S1B clone cl2 | 98.62 (1232) | Radioactive thermal spring, Iran | Heidari et al., 2018 |
| *Thermoleptolyngbya albertanoae* ETS-08 | 98.47 (1439) | Euganean thermal spring, Padova, Italy | Sciuto and Moro, 2016 |
| “*Leptolyngbya* sp.” NgrLPT40 | 97.68 (1424) | Nigrita hot spring, Greece | Bravakos et al., 2016 |
| Thermophilic cyanobacterium tBTRCCn 408 | 97.38 (1375) | Zerka Ma'in thermal spring, Jordan | Oren et al., 2009 |
| Uncultured “*Leptolyngbya* sp.” clone Alla11otu1-1 | 97.25 (1380) | Alla hot spring, Buryatia, Russia | Gaisin et al., 2015 |
| *Thermoleptolyngbya* sp. CENA538 | 97.12 (1414) | Saline-alkaline lake, Nhecolandia, Brazil | Andreote et al., 2014 |
| Uncultured cyanobacterium clone 9B-56 | 97.11 (1451) | Hot spring mat, Tengchong, China | Peng et al., 2013 |
