# Supplementary material for: Description, Taxonomy, and Comparative Genomics of a Novel species, Thermoleptolyngbya sichuanensis sp. nov., Isolated From Hot Springs of Ganzi, Sichuan, China
Source: Front Microbiol. 2021 Sep 10;12:696102. doi: 10.3389/fmicb.2021.696102 (PMC8461337; doi:10.3389/fmicb.2021.696102)
Supplement: Supplementary file 3 [file Table_3.DOCX]

| **Supplementary Table S3** Patristic distance between *Thermoleptolyngbya sichuanensis* PKUAC-SCTA183 and related strains. | | | | | | | | | | | |
| --- | --- | --- | --- | --- | --- | --- | --- | --- | --- | --- | --- |
|  |  | 1 | 2 | 3 | 4 | 5 | 6 | 7 | 8 | 9 | 10 |
| 1 | CP053661 Thermoleptolyngbya sichuanensis PKUAC – SCTA183 |  | 0.03 | 0.07 | 0.09 | 0.14 | 0.28 | 0.36 | 0.39 | 0.36 | 0.33 |
| 2 | DVEA0100000000 Thermoleptolyngbya sp. M55_K2018_002 | 0.03 |  | 0.07 | 0.09 | 0.13 | 0.28 | 0.36 | 0.39 | 0.36 | 0.33 |
| 3 | JACYLP0100000000 Thermoleptolyngbya sp. C42_A2020_037 | 0.07 | 0.07 |  | 0.09 | 0.14 | 0.28 | 0.36 | 0.39 | 0.36 | 0.33 |
| 4 | AP017367 Thermoleptolyngbya sp. O-77 | 0.09 | 0.09 | 0.09 |  | 0.13 | 0.28 | 0.35 | 0.39 | 0.36 | 0.33 |
| 5 | Thermoleptolyngbya oregonensis PCC8501 | 0.14 | 0.13 | 0.14 | 0.13 |  | 0.29 | 0.36 | 0.39 | 0.37 | 0.34 |
| 6 | KL662191 Leptolyngbya sp. JSC-1 | 0.28 | 0.28 | 0.28 | 0.28 | 0.29 |  | 0.36 | 0.4 | 0.37 | 0.34 |
| 7 | LXYR00000000 Phormidesmis priestleyi_BC1401 | 0.36 | 0.36 | 0.36 | 0.35 | 0.36 | 0.36 |  | 0.38 | 0.36 | 0.36 |
| 8 | MUGG00000000 Alkalinema sp. CACIAM70d | 0.39 | 0.39 | 0.39 | 0.39 | 0.39 | 0.4 | 0.38 |  | 0.36 | 0.4 |
| 9 | AP014642 Leptolyngbya boryana dg5 | 0.36 | 0.36 | 0.36 | 0.36 | 0.37 | 0.37 | 0.36 | 0.36 |  | 0.37 |
| 10 | JACJPJ00000000 Oculatella sp. FACHB-28 | 0.33 | 0.33 | 0.33 | 0.33 | 0.34 | 0.34 | 0.36 | 0.4 | 0.37 |  |
| * In the case of *Thermoleptolyngbya oregonensis* PCC8501 following sequences have been used KJ708713, KJ708714, KJ708655, KJ708657, Z19598, DQ010540, EU119379, EU119380, EU119378 | | | | | | | | | | | |

**REFERENCE for Patristic distance:**

[1] Fourment M, Gibbs MJ. PATRISTIC: a program for calculating patristic distances and graphically comparing the components of genetic change. BMC Evol Biol 2006;6:1.
