# Supplementary material for: Description, Taxonomy, and Comparative Genomics of a Novel species, Thermoleptolyngbya sichuanensis sp. nov., Isolated From Hot Springs of Ganzi, Sichuan, China
Source: Front Microbiol. 2021 Sep 10;12:696102. doi: 10.3389/fmicb.2021.696102 (PMC8461337; doi:10.3389/fmicb.2021.696102)
Supplement: Supplementary file 4 [file Table_4.DOCX]

**Table SF 1**. *In silico* DNA-DNA hybridization (DDH) between *Thermoleptolyngbya sichuanensis* PKUAC-SCTA183 and related strains

| Query genome | Reference genome | DDH | Distance | Prob. DDH >= 70% |
| --- | --- | --- | --- | --- |
| PKUAC-SCTA183 | M55_K2018_002 | 60.3 | 0.0511 | 53.4 |
| PKUAC-SCTA183 | C42_A2020_037 | 44 | 0.0872 | 6.67 |
| PKUAC-SCTA183 | O-77 | 38.8 | 0.1036 | 1.99 |
| PKUAC-SCTA183 | AP014642 | 25 | 0.1743 | 0.01 |
| PKUAC-SCTA183 | MUGG01 | 22.8 | 0.1917 | 0 |
| PKUAC-SCTA183 | LXYR01 | 20.9 | 0.2103 | 0 |
| PKUAC-SCTA183 | JSC-1 | 19.7 | 0.2228 | 0 |
| PKUAC-SCTA183 | JACJPJ01 | 19.8 | 0.2215 | 0 |

**REFERENCES for DDH analysis:**

[1] Auch AF, Klenk H-P, Göker M. Standard operating procedure for calculating genome-to-genome distances based on high-scoring segment pairs. Stand Genomic Sci. 2010 2(1): 142–148. doi: 10.4056/sigs.541628.

[2] Goris J, Konstantinidis KT, Klappenbach JA, Coenye T, Vandamme P, Tiedje JM. DNA-DNA hybridization values and their relationship to whole-genome sequence similarities. Int J Syst Evol Microbiol. 2007 57(Pt 1): 81–91. doi: 10.1099/ijs.0.64483-0.
