# Supplementary material for: Description, Taxonomy, and Comparative Genomics of a Novel species, Thermoleptolyngbya sichuanensis sp. nov., Isolated From Hot Springs of Ganzi, Sichuan, China
Source: Front Microbiol. 2021 Sep 10;12:696102. doi: 10.3389/fmicb.2021.696102 (PMC8461337; doi:10.3389/fmicb.2021.696102)
Supplement: Supplementary file 6 [file Table_6.DOCX]

**Table S4** CRISPR-Cas summary of two *Thermoleptolyngbya* strains

| Strain | CRISPR array | Position in genome | CRISPR length (bp) | Direct repeat length (bp) | No. of spacers | Spacer average length (bp) |
| --- | --- | --- | --- | --- | --- | --- |
| A183 | 1 + Cas III-D | 3671960 - 3692685 | 7601 | 35 | 102 | 39.2 |
|  | 2 | 4327377 - 4331068 | 3691 | 35 | 49 | 39.6 |
|  | 3 | 5177256 - 5178540 | 1284 | 35 | 17 | 38.5 |
|  | Total |  | Average | Average | Sum | Total average |
|  | 3 |  | 4192 | 35 | 168 | 39.1 |
| O-77 | 1 | 2574143 - 2590752 | 16609 | 35 | 233 | 36.1 |
|  | 2 | 2590864 - 2592530 | 1666 | 35 | 23 | 35.9 |
|  | 3 | 2592642 - 2602574 | 9932 | 35 | 139 | 36.2 |
|  | 4 | 2603441 - 2604479 | 1038 | 35 | 14 | 36.7 |
|  | 5 + Cas genes | 5224485 - 5227883 | 557 | 35 | 7 | 39.7 |
|  | 6 + Cas III-D | 5285916 - 5307520 | 5071 | 34 | 68 | 39.3 |
|  | 7 | 5307637 - 5315948 | 8311 | 34 | 113 | 39.3 |
|  | Total |  | Average | Average | Sum | Total average |
|  | 7 |  | 6169 | 34.7 | 597 | 37.6 |
