# Supplementary material for: Description, Taxonomy, and Comparative Genomics of a Novel species, Thermoleptolyngbya sichuanensis sp. nov., Isolated From Hot Springs of Ganzi, Sichuan, China
Source: Front Microbiol. 2021 Sep 10;12:696102. doi: 10.3389/fmicb.2021.696102 (PMC8461337; doi:10.3389/fmicb.2021.696102)
Supplement: Supplementary file 7 [file Data_Sheet_1.PDF]

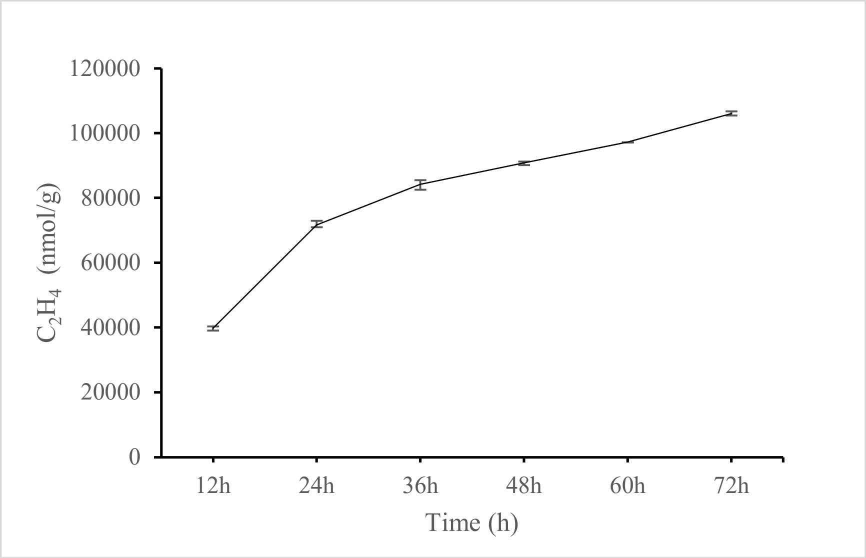


**Supplementary Figure S1** Nitrogenase activity of cell suspensions of *Thermoleptolyngbya sp.* A183 represented as a acetylene reduction proxy assay. Assay presents nmol/$g_{cells}$ of ethylene released from the strain grown in nitrogen-free BG-11 medium during 72 h, 45°C, 30 µmol m $^{2}$ s $^{1}$ gas composition Ar/$N_2$/$CO_2$ = 90/9/1 [v/v/v %].
