# Supplementary figures and images for: Description, Taxonomy, and Comparative Genomics of a Novel species, Thermoleptolyngbya sichuanensis sp. nov., Isolated From Hot Springs of Ganzi, Sichuan, China
Source: Front Microbiol. 2021 Sep 10;12:696102. doi: 10.3389/fmicb.2021.696102 (PMC8461337; doi:10.3389/fmicb.2021.696102)

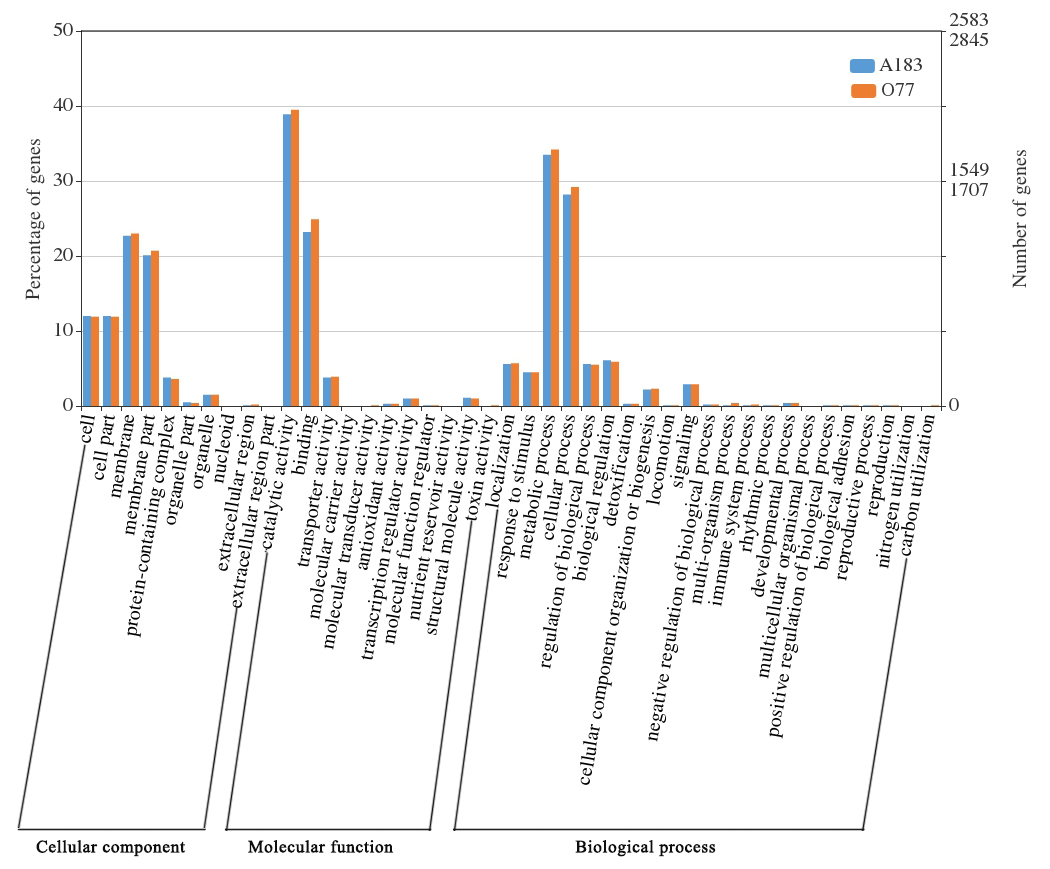

Supplement: Supplementary file 9 [file Image_1.JPEG]

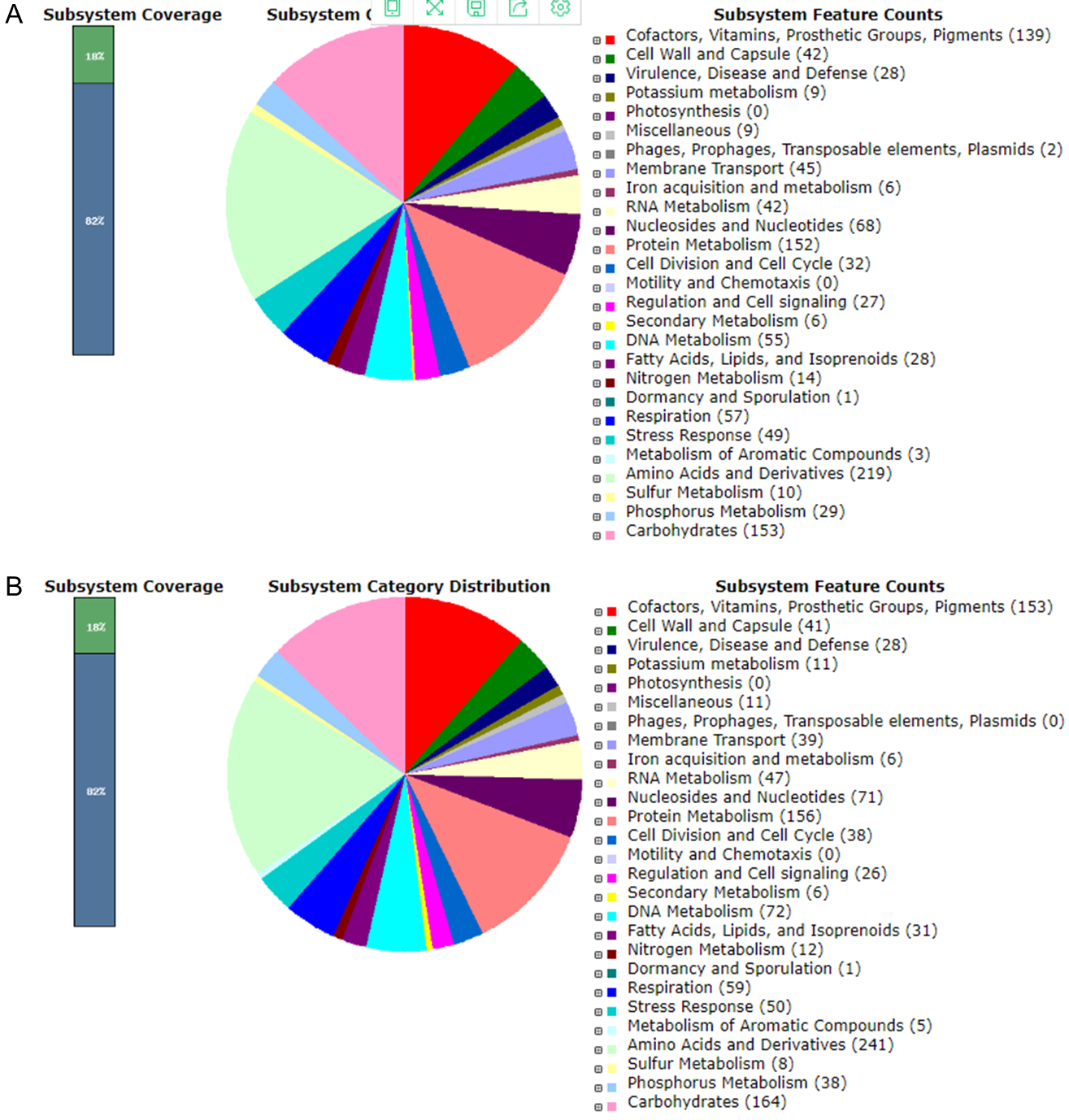

Supplement: Supplementary file 11 [file Image_3.TIFF]

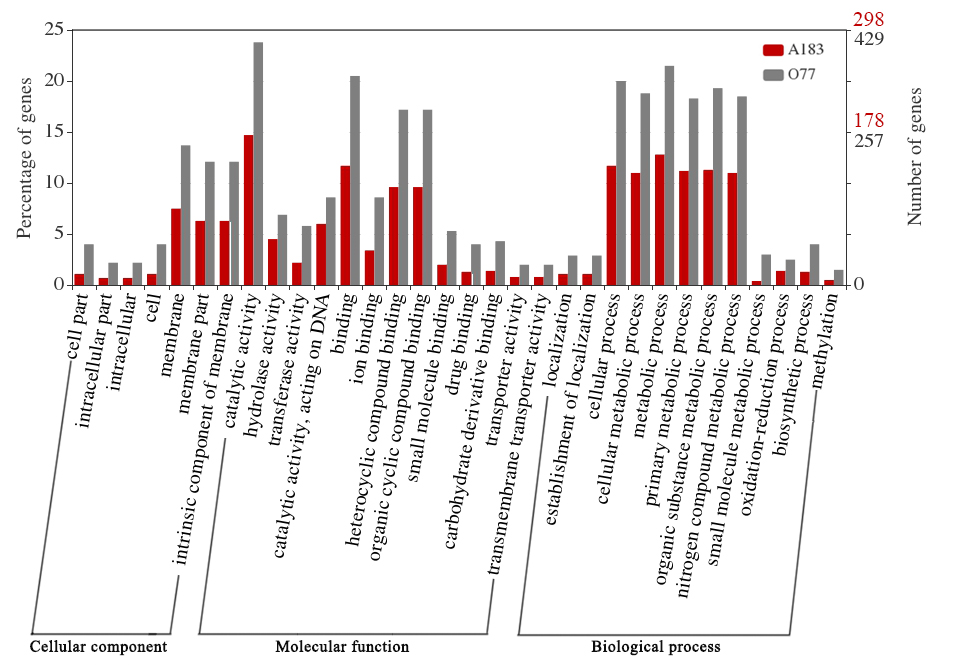

Supplement: Supplementary file 12 [file Image_4.JPEG]
