# Supplementary material for: Description, Taxonomy, and Comparative Genomics of a Novel species, Thermoleptolyngbya sichuanensis sp. nov., Isolated From Hot Springs of Ganzi, Sichuan, China
Source: Front Microbiol. 2021 Sep 10;12:696102. doi: 10.3389/fmicb.2021.696102 (PMC8461337; doi:10.3389/fmicb.2021.696102)
Supplement: Supplementary file 8 [file Data_Sheet_2.PDF]

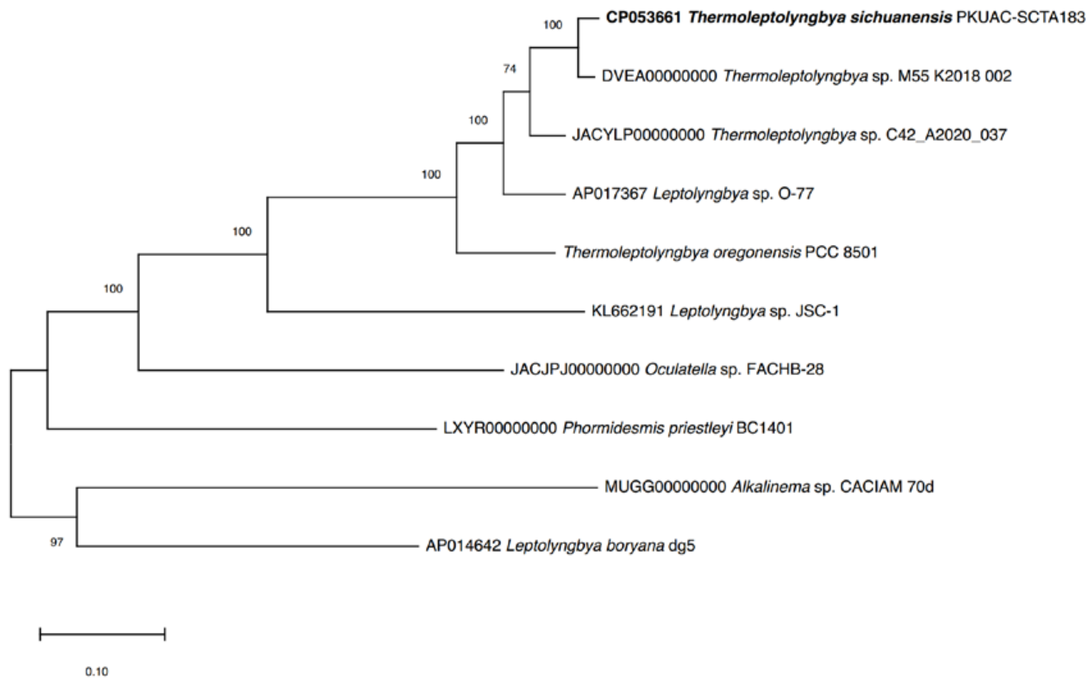

Supplementary Figure S4 Maximum-Likelihood tree based on concatenated sequences (9,697 bp) of twelve genes: cpcB, cpcA, gyrA, nir, nrtA, nrtB, nrtC, nrtD, rbcL, rbcS, rbcX, rpoC

Method used:

The bootstrap consensus was inferred from 1000 replicates. Phylogenetic analysis was conducted using MEGA X software (www.megasoftware.net) with the Maximum-Likelihood method and the General Time Reversible, with Gamma distribution as the best nucleotide substitution model. Phylogenetic analysis was carried out on the basis of sequences retrieved from genomes of *Thermoleptolyngbya* and closely related *Leptolyngbya* strains that were available at GenBank (in May 2021), whereas, in the case of *Thermoleptolyngbya oregonensis* PCC8501 following sequences have been used KJ708713, KJ708714, KJ708655, KJ708657, Z19598, DQ010540, EU119379, EU119380, EU119378.
